# An Endemic Region of Thiamine-Responsive Megaloblastic Anemia Caused by an *SLC19A2* c.1223+1G>A Founder Mutation

**DOI:** 10.3390/ijms27146274

**Published:** 2026-07-14

**Authors:** Medina Gurzhikhanova, Sergei Fomenko, Nikolay Chekanov, Olga Musharova, Tatyana Salimova, Olga Goronkova, Ruslan Abasov, Elena Raykina, Rena Zinchenko, Margarita Sharova, Fizaliia Khisamieva, Evgeny Imyanitov, Anna Sokolenko, Evgeny Klimuk, Alexey Maschan, Konstantin Severinov, Michael Maschan

**Affiliations:** 1 Dmitry Rogachev National Medical Research Center of Pediatric Hematology, Oncology, and Immunology, 117198 Moscow, Russia; medina.gurzhihanova@dgoi.ru (M.G.); tatyana.salimova@dgoi.ru (T.S.); olga.goronkova@dgoi.ru (O.G.); ruslan.abasov@dgoi.ru (R.A.); elena.raykina@dgoi.ru (E.R.); amaschan@mail.ru (A.M.); 2 Biotech Campus LLC, 117437 Moscow, Russia; sfomenko@biotc.ru (S.F.); nchekanov@biotc.ru (N.C.); musharova_olga@mail.ru (O.M.); eklimuk@biotc.ru (E.K.); 3 Research Centre for Medical Genetics, 115522 Moscow, Russia; renazinchenko@mail.ru (R.Z.); rsharova221195@gmail.com (M.S.); 4 The State Autonomous Healthcare Institution “Children’s Republican Clinical Hospital of the Ministry of Health of the Republic of Tatarstan”, 420064 Kazan, Russia; kh.f.f@yandex.ru; 5 Department of Tumor Growth Biology, N.N. Petrov Institute of Oncology, 197758 Saint Petersburg, Russia; evgeny@imyanitov.spb.ru (E.I.); annasokolenko@mail.ru (A.S.); 6 Department of Medical Genetics, St.-Petersburg Pediatric Medical University, 194100 Saint Petersburg, Russia

**Keywords:** thiamine-responsive megaloblastic anemia, *SLC19A2*, founder effect, Rogers syndrome, whole-genome sequencing

## Abstract

Thiamine-responsive megaloblastic anemia (TRMA) is a rare autosomal recessive disorder caused by biallelic loss of function variants in the *SLC19A2* gene. It typically presents with a triad of megaloblastic anemia, diabetes mellitus, and sensorineural deafness. In this work, we analyzed ten children with suspected TRMA: nine exhibited the full triad and one, a younger sibling of a patient with full triad, did not develop hearing loss by the age of 18 months. All patients became transfusion-independent on high-dose thiamine therapy. Whole-genome sequencing identified homozygosity for the canonical splice variant *SLC19A2* c.1223+1G>A in eight patients. One patient was homozygous for a known *SLC19A2* c.196G>T variant, and the other was compound heterozygous for two novel variants, c.120C>G and c.584T>C. All patients with the *SLC19A2* c.1223+1G>A variant were ethnic Ingush. In the reference Ingush cohort, 9/328 unrelated adults were c.1223+1G>A carriers (heterozygous carrier frequency 2.7%; carrier frequency ≈ 1/36), and shared a 2.3 Mb *ATP1B1*–*FMO2* haplotype on chromosome 1, demonstrating a strong founder effect. These findings identify Ingushetia as a new TRMA-endemic region and support targeted *SLC19A2* screening and early thiamine therapy in patients with macrocytic anemia and diabetes of unclear origin in this population.

## 1. Introduction

Thiamine-responsive megaloblastic anemia (TRMA) is a rare autosomal recessive disorder characterized by the triad of megaloblastic anemia, diabetes mellitus, and sensorineural deafness most frequently arising in childhood. First described in the twentieth century [1], TRMA reflects the essential role of thiamine in cellular metabolism, especially in the development and function of hematopoietic, pancreatic, and auditory systems. The disorder is caused by biallelic mutations in the *SLC19A2* gene, which encodes the high-affinity thiamine transporter 1 (THTR-1), a protein critical for thiamine uptake in sensitive tissues [2]. Loss of function mutations in *SLC19A2* result in cellular thiamine deficiency even in the presence of normal dietary intake, underpinning the core pathology of the syndrome.

Several *SLC19A2* mutations leading to TRMA have been documented worldwide.

While TRMA remains exceptionally rare in most parts of the world, a handful of regional clusters have been reported—often in areas where kinship networks or geographic isolation have limited genetic diversity and facilitated the propagation of founder mutations [3]. In this context, the identification of a new founder mutation generating an endemic region for TRMA offers an opportunity both to advance the molecular understanding of the disease and to elucidate its historical genesis within specific populations.

In this work, we present ten clinically and genetically ascertained TRMA cases. Eight of these cases shared an identical *SLC19A2* nucleotide substitution c.1223+1G>A and were confirmed via haplotype and kinship analysis to be derived from a single ancestral origin in the Ingush population.

## 2. Results

### 2.1. Clinical Presentations of Patients with Suspected TRMA

All patients, except for one, manifested a classical triad of thiamine-responsive megaloblastic anemia: early-onset anemia, diabetes mellitus, and sensorineural hearing loss. Patient 5, a younger sibling of patient 3, had not developed deafness at last assessment (1.5 years of age) but remains at risk for subsequent onset, in line with the progressive nature of hearing loss in TRMA (Table 1).

Anemia was the earliest documented symptom for most cases, presenting between 1 month and 5 years of age. Several children initially received alternative hematological diagnoses (e.g., myelodysplastic neoplasms (MDS) or inherited bone marrow failure) prior to definitive molecular identification. Diabetes mellitus was uniformly present, with all patients developing hyperglycemia between infancy and early childhood (ages 11 months to 8 years). All patients (except patient 5) initially required ongoing insulin therapy for metabolic control, before they got their molecular diagnosis. Sensorineural hearing loss occurred in nine patients, with clinical onset range between 8 months and 2.5 years in seven cases; age of onset was not available for two individuals due to incomplete clinical records.

Ophthalmological abnormalities, including optic atrophy and decreased visual acuity, were observed in four children (patients 4, 6, 7, and 8). Cardiac defects—supraventricular tachycardia, mitral valve insufficiency, or patent ductus arteriosus—were present in three patients (patients 1, 2, and 4), with patient 4 exhibiting both ocular and cardiac involvement. These findings illustrate the extended spectrum of TRMA beyond the classical triad, encompassing additional organ involvement that carries implications for multidisciplinary management.

The overall disease course was characterized by the persistence of anemia and diabetes mellitus, with long-term transfusion independence achievable after initiation of thiamine supplementation for all patients. However, the course of diabetes showed greater variability: while all patients initially required insulin, six achieved stable insulin independence following long-term thiamine supplementation, whereas four (patients 1, 4, 6, and 7) remain insulin-dependent. Hearing loss, however, remained refractory to treatment and continued to progress regardless of thiamine therapy. Regular follow-up allowed monitoring of metabolic status, auditory and ophthalmic function, and cardiac health, all of which are crucial for optimizing outcomes in affected individuals.

### 2.2. Results of Genetic Testing

**Figure 1:**
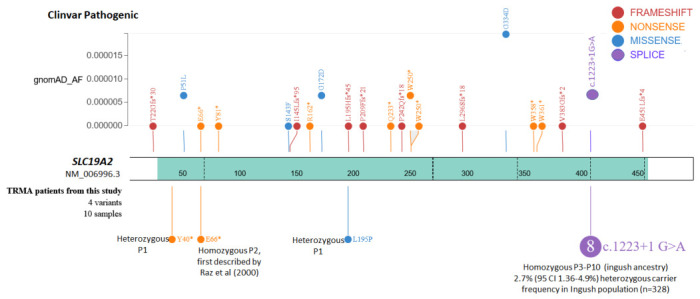
Distribution of *SLC19A2* pathogenic variants along the protein sequence. * is the standard sign for describing nonsense variant by protein as explained by color-coded legend. The lollipop plot displays all ClinVar-classified pathogenic variants in *SLC19A2* (NM_006996.3) in the upper panel and the four variants identified in the present cohort in the lower panel. Each circle represents a single variant; the vertical stem height in the upper panel is proportional to the gnomAD v4.0 allele frequency (gnomAD_AF) of the respective variant. Variant classes are color-coded: frameshift (red), nonsense/stop-gained (orange), missense (blue), and splice-site (purple). The teal bar indicates the single coding region of the THTR-1 protein (residues 1–496 of the canonical isoform), with amino acid position numbers shown along the horizontal axis. Upper panel (ClinVar Pathogenic): twenty-three previously reported pathogenic variants are shown. The missense variant p.(Gly334Asp) exhibits the highest gnomAD_AF among all displayed variants (~0.000020), while the majority of pathogenic alleles are absent or present at frequencies below 0.000010 in the general population. Lower panel (TRMA patients from this study, 4 variants, 10 samples): Patient 1 (Altai region) harbored two heterozygous variants—p.(Tyr40Ter) (nonsense) and p.(Leu195Pro) (missense)—in trans, constituting a novel compound heterozygous genotype. Patient 2 (Central Asian origin) was homozygous for p.(Glu66Ter), a nonsense variant first described by Raz et al. in consanguineous families [4]. Patients 3–10 (all of Ingush ancestry) were homozygous for the canonical splice-site variant c.1223+1G>A; this variant was detected in 8 of 10 (80%) patients and carries a heterozygous carrier frequency of 2.7% (95% CI 1.36–4.9%) in the Ingush reference population (*n* = 328), consistent with a strong founder effect.

Definitive diagnosis of TRMA was established in all ten patients by whole-genome sequencing (WGS) (Figure 1). At the time of genetic analysis, the ages of patients were between 11 months and 16 years.

Patient 1, originating from the Altai region, was compound heterozygous for *SLC19A2* variants c.120C>G and c.584T>C, neither of which has been previously described in the literature or reported in association with TRMA. Each variant was observed as a single allele in gnomAD, indicating extremely low population frequencies consistent with pathogenicity. The c.120C>G substitution introduces a premature stop codon at position 40 p.(Tyr40Ter) that should commit the transcript to nonsense-mediated decay (NMD); this variant was therefore classified as likely pathogenic based on its null effect and concordant phenotype. In trans, the c.584T>C variant results in a missense change p.(Leu195Pro) affecting a highly conserved residue within the transporter; multiple in silico prediction tools, including CADD (30, Pathogenic Moderate) and AlphaMissense (0,97 Pathogenic Moderate), support the deleterious effect on protein structure and function.

Patient 2 of Central Asian origin was found to harbor a homozygous *SLC19A2* c.196G>T variant, a known pathogenic nonsense mutation previously described by Raz and colleagues [4]. It is considered a null allele as it introduces a premature termination codon p.(Glu66Ter), that should commit the transcript to NMD. The c.196G>T p.(Glu66Ter) variant has been reported in seven independent TRMA families, six from Pakistan [4,5] and one from India [6], as summarized in a recent mutation-update study [3]. All Pakistani patients described so far were born to consanguineous parents, consistent with autosomal recessive inheritance in populations with a high rate of intra-familial marriage.

Patients 3 to 10 were homozygous for the *SLC19A2* c.1223+1G>A mutation. This mutation affects the invariant guanine at the +1 position of the donor splice site located at intron 4 of the *SLC19A2* gene. This position is required for recognition by the spliceosome during pre-mRNA processing. Disruption of this site by the G>A substitution at the exon-intron boundary is predicted to lead to incorrect mRNA splicing by multiple algorithms (e.g., MaxEntScan, NNSplice, SpliceAI). As a result, a part of the following intron is abnormally included in the mature transcript, which extends the exon and introduces a premature stop codon 8 codons downstream of the aberrant splice site. This should trigger NMD and result in the loss of the functional *SLC19A2* transcript. Consequently, homozygous individuals have no THTR-1 [7,8]. This specific mutation was previously described only once by Raz and colleagues [4], in the first set of *SLC19A2* mutations identified as causative for TRMA. The clinical characteristics of the only known patient with *SLC19A2* c.1223+1G>A, an 8-year-old Lebanese girl, were previously described by Bazarbachi et al. [9]. She originated from a consanguineous family, which reflects the autosomal recessive inheritance pattern of TRMA syndrome and emphasizes the genetic risk associated with consanguinity. This patient presented with the classical triad characteristic of the syndrome: early-onset megaloblastic anemia necessitating multiple transfusions, sensorineural deafness detected in infancy requiring hearing aids, and childhood-onset diabetes mellitus.

### 2.3. Results of Genetic Epidemiology Study

All patients with the *SLC19A2* c.1223+1G>A mutation were from Ingushetia, an autonomous republic in the Northern Caucasus that is home to approximately 500 thousand people. In the control cohort of 328 unrelated ethnically Ingush adults, whole-genome sequencing identified 9 heterozygous carriers of *SLC19A2* c.1223+1G>A, corresponding to heterozygous carrier frequency of 2.7% and estimated carrier frequency of ~1 in 36 individuals. To confirm the exceptionally high frequency of the *SLC19A2* c.1223+1G>A allele in Ingush population, an independent collection of DNA samples obtained from adult oncological patients from Ingushetia was surveyed by allele-specific PCR. Five *SLC19A2* c.1223+1G>A heterozygotes were identified in 259 samples from unrelated subjects.

Kinship and haplotype analysis demonstrated that all carriers shared a common 2.3-Mb haplotype on chromosome 1 (chr1:168,981,142–171,250,829), consistent with descent from a single ancestral founder. The shared segment extends from *ATP1B1* and encompasses *SLC19A2* together with 19 additional coding genes, terminating at *FMO2*. Within this interval, other genes with OMIM-annotated disease associations include F5, *GORAB*, *PRRX1*, and *FMO3*, indicating that this founder haplotype harbors multiple loci with potential clinical relevance, although no additional pathogenic variants were detected in *SLC19A2* c.1223+1G>A carriers from our cohort.

**Figure 2:**
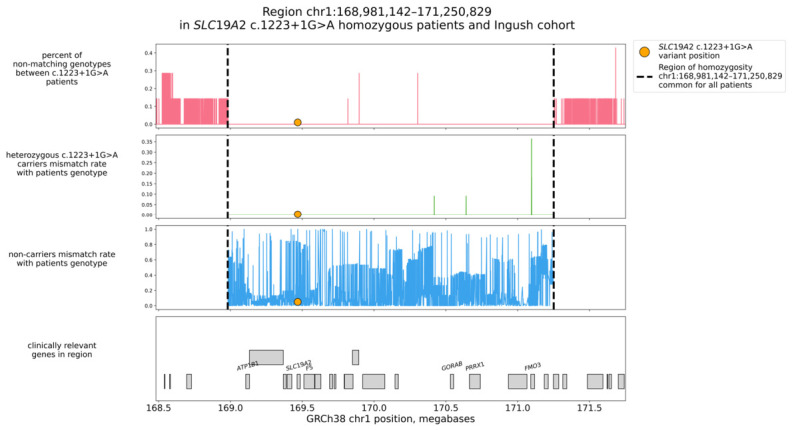
Haplotype analysis of the *SLC19A2* c.1223+1G>A founder region in homozygous patients and the reference Ingush cohort. Multi-panel plot of the genomic region chr1:168,981,142–171,250,829 (GRCh38). The x-axis represents physical position on chromosome 1 in megabases. Top panel (pink): percent of non-matching genotypes between the eight homozygous *SLC19A2* c.1223+1G>A patients (Patients 3–10), showing near-zero discordance across the ~2.3-Mb core haplotype (dashed vertical lines). Second panel (green): genotype mismatch rate of heterozygous *SLC19A2* c.1223+1G>A carriers from the Ingush reference cohort against the patient consensus haplotype; carriers show near-zero mismatch across the founder interval, confirming identity by descent. Third panel (blue): genotype mismatch rate of non-carriers from the same Ingush cohort against the patient haplotype, demonstrating high and variable discordance throughout the region, reflecting normal population-level haplotype diversity. The orange circle in each panel marks the position of the c.1223+1G>A splice-site variant within *SLC19A2*. Bottom panel: gene map of the region (GRCh38 annotation), with *SLC19A2* indicated among neighboring clinically relevant genes (*ATP1B1*, *F5*, *SLC19A2*, *PRRX1*, *FMO3*). Dashed vertical lines demarcate the boundaries of the shared founder haplotype (chr1:168,981,142–171,250,829).

The genotype mismatch rate relative to the consensus haplotype of homozygous patients 3–10 was computed across a ~2.3 Mb window on chromosome 1 (chr1:168,981,142–171,250,829; GRCh38) (Figure 2). Among the eight homozygous patients, pairwise genotype comparison revealed near-zero mismatch rates across the entire interval (mean genotype concordance across region—99.98%), with only rare discordant positions at the flanking boundaries, confirming that all patients carry an identical-by-descent haplotype block of approximately 2.3 Mb. Heterozygous *SLC19A2* c.1223+1G>A carriers identified in the Ingush reference cohort similarly demonstrated a near-zero mismatch rate with the patient haplotype across the same interval, indicating that they too carry the identical founder allele on one chromosome. In contrast, non-carriers from the same Ingush cohort showed high and variable mismatch rates throughout the region, reflecting the expected population-level haplotype diversity. The sharp contrast between the uniformly conserved haplotype in carriers and the heterogeneous background in non-carriers provides unambiguous evidence that all *SLC19A2* c.1223+1G>A alleles in the Ingush population are identical by descent, consistent with a single founder event (Figure 2).

**Figure 3:**
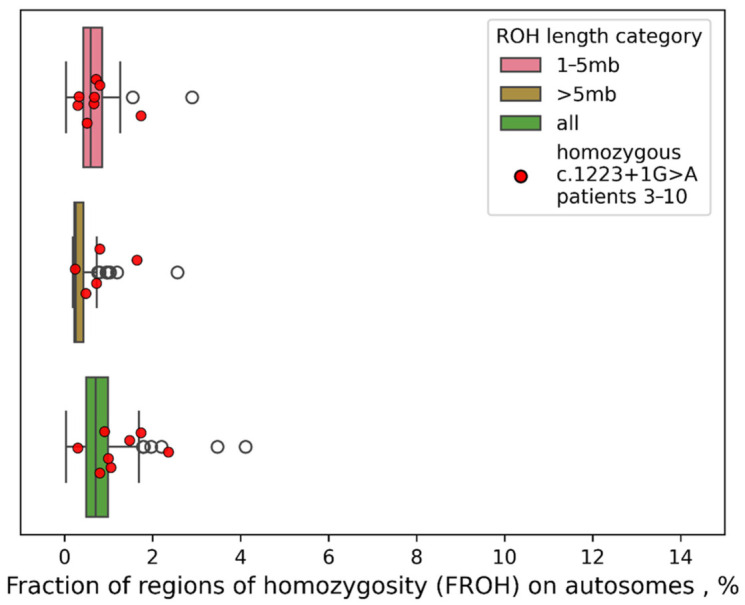
ROH length distribution in the Ingush population and homozygous *SLC19A2* c.1223+1G>A patients. Boxplots show the distribution of FROH in the Ingush reference population (*n* = 328) stratified by ROH length category: short (1–5 Mb, pink), long (>5 Mb, olive), and all ROH combined (green). Red dots represent individual values for homozygous *SLC19A2* c.1223+1G>A patients (Patients 3–10). Whiskers extend to 1.5 × IQR; open circles indicate outliers. Patient FROH values fall within the population distribution in all three categories, indicating the absence of elevated background consanguinity.

To assess the degree of consanguinity in both the patient cohort and the Ingush reference population, runs of homozygosity (ROH) analysis was performed genome-wide for all sequenced individuals. The fraction of the autosomal genome covered by ROH (FROH) was calculated separately for short (1–5 Mb) and long (>5 Mb) segments, as well as cumulatively for all ROH (Figure 3). For each individual, FROH was used as the genomic inbreeding coefficient (F). The FROH values of all eight homozygous SLC19A2 c.1223+1G>A patients fell below 5% in every category—short, long (>5 Mb), and all ROH combined—and within the distribution of the general Ingush reference cohort, below the genome-wide homozygosity level (~5%, corresponding to roughly second-cousin or closer unions) considered indicative of recent consanguinity [10]. These data indicate that the patients and the Ingush controls were not derived from consanguineous families and that the homozygosity observed at the *SLC19A2* locus in the patients reflects identity by descent from a shared population-level founder allele rather than recent parental relatedness. Although long contiguous stretches of homozygosity can recur at the same locus across unrelated individuals without indicating parental consanguinity [11,12,13], a founder allele is distinguished by the co-occurrence of a single locus-specific homozygous segment with a normal genome-wide homozygosity burden. Accordingly, the conserved 2.3-Mb chromosome-1 block encompassing *SLC19A2* recurs in heterozygous form among unrelated Ingush carriers (Figure 2), while patients’ genome-wide FROH values remain within the normal Ingush range (Figure 3).

**Figure 4:**
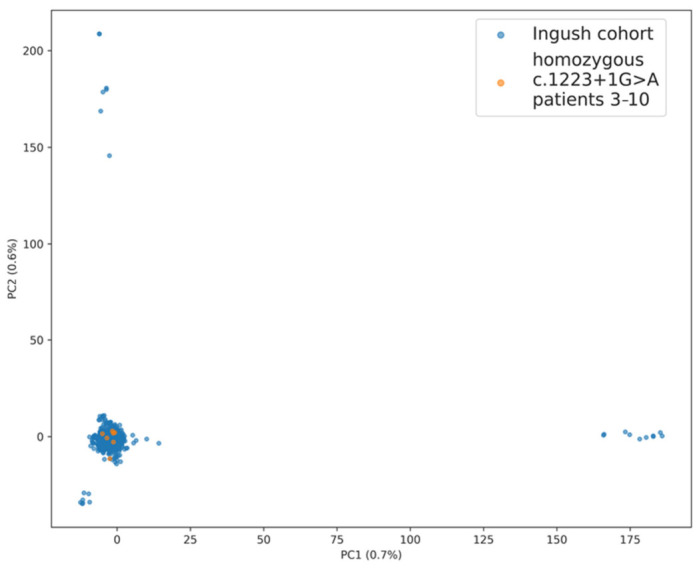
Principal component analysis of the Ingush cohort and homozygous *SLC19A2* c.1223+1G>A patients. Scatter plot of the first two principal components derived from genome-wide SNP data. Blue dots represent individuals from the Ingush reference cohort (*n* = 328); orange dots represent homozygous *SLC19A2* c.1223+1G>A patients (Patients 3–10). PC1 explains 0.7% and PC2 explains 0.6% of total variance. All eight patients cluster within the central Ingush reference cloud, confirming Ingush genetic ancestry and ruling out population stratification as a confounding factor.

Principal component analysis (PCA) was performed on genome-wide SNP data to verify the reported Ingush ancestry of the eight homozygous patients and to confirm that their genetic background was concordant with the Ingush reference cohort. In the PC1–PC2 projection, all eight patients clustered tightly within the main Ingush reference cloud, with PC1 accounting for 0.7% and PC2 for 0.6% of total variance. This co-localization demonstrates that the patients are genetically indistinguishable from the Ingush population and rules out the possibility of cryptic non-Ingush ancestry that could confound the founder-effect interpretation (Figure 4).

**Figure 5:**
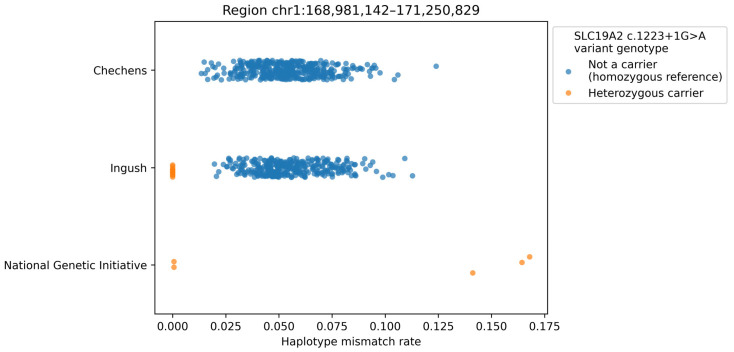
The haplotype mismatch rates, relative to the consensus Ingush c.1223+1G>A haplotype, in three groups: Ingush WGS controls, Chechen WGS controls, and heterozygous carriers from the “National Genetic Initiative” (NGI, general Russian population). Ingush heterozygous carriers cluster near zero mismatch, indicating an almost identical shared 2.3-Mb haplotype, whereas Chechen individuals show uniformly higher mismatch values, consistent with the absence of the Ingush founder haplotype. Among NGI carriers, two individuals demonstrate low mismatch rates comparable to Ingush carriers and three display substantially higher mismatch rates, indicating that their c.1223+1G>A alleles arose on distinct non-Ingush haplotypic backgrounds.

As a next step, we analyzed WGS data from a cohort of 391 unrelated Chechen individuals and did not detect a single case of a *SLC19A2* c.1223+1G>A heterozygote (Figure 5). Available data indicate that the Chechen and Ingush populations separated approximately 1400 years ago [14], implying that the extremely rare *SLC19A2* c.1223+1G>A allele was either present in one of the members of the Ingush population founders or had arisen shortly after the two populations became separate and then spread among the Ingush. The length of the *SLC19A2* c.1223+1G>A carrying haplotype chr1:168,981,142–171,250,829 (2.3-Mb) is consistent with either scenario.

А survey of ~50,000 genomes of healthy volunteers from the “National Genetic Initiative” (NGI) in the Biotech Campus database [15] predominantly self- identifying as ethnic Russians, Tatars, and Bashkirs revealed five *SLC19A2* c.1223+1G>A heterozygous carriers (Figure 5). Two volunteers, according to a self-questionnaire, had mixed North Caucasian ancestry, and the c.1223+1G>A allele in both individuals was carried on the Ingush founder haplotype. The remaining three carriers identified themselves as ethnic Russians and their c.1223+1G>A alleles were not part of the Ingush haplotype, indicating independent occurrence of the mutation.

## 3. Discussion

In populations with high endogamy or consanguinity typical to many communities in the Caucasus [16,17] understanding the molecular basis of disease clusters can dramatically improve the cost-effectiveness and accuracy of genetic testing and intervention programs. Isolated reports have identified regional clusters of phenylketonuria, familial Mediterranean fever, and hereditary sensorineural deafness attributable to population-specific founder events in the Russian Caucasus [16,17,18]. Despite these advances, this highly diverse region is underrepresented in global rare disease research.

In this study, we describe the largest cohort of TRMA patients from a single country and single region who had the same mutation *SLC19A2* c.1223+1G>A. The therapeutic experience in this cohort underscores the importance of early molecular recognition. All patients received thiamine supplementation and became transfusion-independent, confirming the reversibility of the hematologic component with adequate replacement. However, diabetes outcomes were heterogeneous: those who started thiamine early in the disease course are currently insulin-independent, whereas four patients, in whom thiamine therapy was initiated later in life, remain dependent on insulin. This observation aligns with published data indicating that timely thiamine replacement in TRMA can preserve or partially restore β-cell function, whereas delayed treatment is associated with persistent insulin dependence [19].

Ingushetia is a small republic in the North Caucasus of southwestern Russia notable for its unique cultural traditions, mountainous geography, and genetic heritage. Despite centuries of interaction with neighboring populations, the Ingush society maintains many practices conducive to the propagation of founder effects, including clan-based kinship structures and periods of forced displacement that have historically reduced genetic diversity [20]. Historical patterns of endogamy, migration, and local population bottlenecks make Ingushetia a uniquely rich source for genetic epidemiology studies [18,21]. The frequency of the *SLC19A2* c.1223+1G>A allele in the Ingush population may be as high as 2.7%, which explains the unusually high number of TRMA patients we observe.

As of today, no *SLC19A2* mutation had previously been proven to have a founder effect in TRMA pathogenesis. The *SLC19A2* c.196G>T p.(Glu66Ter) nonsense variant had previously emerged as one of the most frequently reported pathogenic alleles in TRMA and, importantly, the only one described in such a large number of families from a single country [3]. Available data indicate that six unrelated families from Pakistan carrying this variant represented the largest group of TRMA kindreds worldwide attributable to a single *SLC19A2* mutation to date, suggesting marked local enrichment rather than a globally common allele. This pattern, together with the observation that all reported Pakistani patients with c.196G>T were born to related parents, is highly suggestive of an underlying founder effect in this population, although no formal haplotype or coalescent analyses have yet been undertaken to confirm this hypothesis [4,5]. Consistent with a geographically restricted founder mutation, population-scale datasets show only a single c.196G>T allele within the South Asian cohort of gnomAD, underscoring the rarity of this variant in the general population and supporting the view that its high clinical visibility in Pakistan reflects local demographic history and consanguinity, rather than high background frequency.

For single-gene disorders such as TRMA, the presence of a local founder mutation not only facilitates rapid diagnosis, but also informs carrier screening, genetic counseling, therapeutic decision-making, and resource allocation. Identification of a *SLC19A2* founder mutation in the Ingush population has immediate clinical implications. In practical terms, an Ingush patient presenting with reduced hemoglobin on complete blood count and marked macrocytosis (MCV above 110 fL) should prompt consideration of TRMA; in such cases, empiric initiation of high-dose thiamine supplementation and targeted analysis (Sanger sequencing or allele-specific PCR) for the c.1223+1G>A variant are reasonable first-line steps, given the high local carrier frequency and the strong founder effect.

## 4. Materials and Methods

### 4.1. Patients Cohort

Ten children with suspected TRMA were enrolled from the Dmitry Rogachev National Research Center of Pediatric Hematology, Oncology and Immunology, Moscow. The median age of patients at the time of diagnosis was 2 years (1 year–16 years), 7 of them were male and 3 female. All were evaluated and followed by pediatric hematology and endocrinology teams, and detailed clinical data (ethnic origin, family history, consanguinity, age at onset of anemia, diabetes and deafness, initial working diagnosis, and associated ophthalmologic and cardiac features) were extracted from medical records using a standardized case report form. Peripheral blood was collected in EDTA tubes, and genomic DNA was isolated according to institutional protocols. WGS was performed as a first-line diagnostic tool in all ten patients to establish a molecular diagnosis.

### 4.2. Reference Group for Genetic Epidemiology Studies

A control group of 328 unrelated ethnically Ingush adults was recruited from the general population of the Republic of Ingushetia. The cohort comprised 186 females (57%) and 142 males (43%), all permanently residing in Ingushetia and aged 19–78 years (median 38 years), without reported serious chronic illnesses at the time of inclusion. Peripheral venous blood was collected in EDTA tubes, and genomic DNA was extracted for whole-genome sequencing.

A control group of 391 unrelated ethnically Chechen adults was recruited from the general population of the Republic of Chechnya. The cohort comprised 164 females (42%) and 227 males (58%), all permanently residing in Chechnya and aged 20–77 years (median 36 years), without reported serious chronic illnesses at the time of inclusion. Peripheral venous blood was collected in EDTA tubes, and genomic DNA was extracted for whole-genome sequencing.

### 4.3. Genetic Laboratory Testing

#### 4.3.1. Whole-Genome Sequencing (WGS)

WGS was performed using the DNBSEQ-T7RS platform. Genomic DNA was isolated from the blood-containing plates using a MGIEasy Magnetic Beads Genomic DNA Extraction Kit (“MGI”, Shenzhen, China) according to the manufacturer’s protocol. The extracted DNA was quantified using a QubitTM dsDNA Quantification Assay Kit (“ThermoFisherScientific”, Waltham, MA, USA). Each gDNA sample (1000 ng) was used to construct a genomic DNA library using the MGIEasy Fast PCR-FREE FS DNA Library Prep Set V2.0 (“MGI”, Shenzhen, China) according to the manufacturer’s instructions. DNA was fragmented by enzymatic fragmentation using magnetic beads. DNA end-repair and adapter ligation were conducted using the MGIEasy UDB PF Adapters-96 Kit (“MGI”, Shenzhen, China). The products were run on a 4200 TapeStation using the Agilent D1000 ScreenTape (“Agilent”, Santa Clara, CA, USA) to assess the size distribution of the libraries. They were also quantified using a QubiTM dsDNA Quantification Assay Kit. The PCR products were circularized and 75 fmol of ssCirDNA was amplified using rolling-circle amplification to generate DNA nanoball-based libraries, which were loaded onto a DNBSEQ-T7RS sequencing flow cell with a DNBSEQ-T7RS High-throughput Sequencing Kit (“MGI”, Shenzhen, China). The library was run on a DNBSEQ-T7RS platform at paired-end 150 bp reads.

#### 4.3.2. Validation Study

For the validation study of the population frequency of the *SLC19A2* c.1223+1G>A variant, we utilized the collection of blood DNA samples, which was available in the N.N. Petrov Institute of Oncology (St.-Petersburg, Russia). This group included 259 unrelated oncological patients (249 women and 10 men; median age: 53 years (range: 21–83 years) of Ingush ethnic origin, who resided in the Republic of Ingushetia and were referred to the above Institute mainly for the diagnostics of hereditary cancer syndromes. The presence of the *SLC19A2* c.1223+1G>A allele was analyzed by allele-specific PCR. Amplification was performed in standard conditions using primers 5′-GAATCATCTACATGTTACTC-3′ and 5′-CTGTTACAATTTTTCCTAAGG-3′, and allele discrimination was achieved with fluorescently labelled probes FAM-CACGATAGCAACGTATGTATTTTGGC-BHQ1 (wild-type) and SLC19A2-MUT HEX-CACGATAGCAACATATGTATTTTGGC-BHQ1 (mutant).

### 4.4. Bioinformatic Analysis

**Preprocessing:** Low-quality read ends and adapters were trimmed using cutadapt v.4.2 [22] with parameters “--trim-n --quality-cutoff 30,30 --error-rate 0.1 --times 99 --minimum-length 0 --pair-filter both --interleaved”. Read pair mapping was performed using BWA-mem v.0.7.17 [23] with parameters “-k 30 -K 100,000,000 -Y” to the human reference genome GRCh38 and sorted with samtools v.1.16.16 [24]. The quality of the bases in the reads was checked using FastQC v.0.11.9 (www.bioinformatics.babraham.ac.uk/projects/fastqc/, accessed on 10 December 2025) software. All cases had mean depth of coverage more than 30x.

**Variant calling and annotation:** Variant calling was processed with DeepVariant algorithm under Clara Parabricks v.4.0.0 [25] with parameters “--disable-use-window-selector-model --normalize-reads --track-ref-reads --min-mapping-quality 10”. The identified genetic variants were designated in accordance with the HGVS nomenclature v.21.0.0 (https://hgvs-nomenclature.org, accessed on 3 December 2025), relative to the MANE Select reference transcript NM_006996.3 (*SLC19A2*); predicted protein consequences are given in parentheses to indicate that they were inferred from genomic sequence rather than confirmed at the RNA or protein level. The population frequencies of these variants were obtained from GnomAD v4.1.1 (http://gnomad.broadinstitute.org/, accessed on 10 December 2025) data. The clinical (diagnostic) significance of the variants was evaluated using the OMIM database (http://omim.org, accessed on 10 December 2025) and open publications. Lollipop plots were generated using ProteinPaint [26].

**Regions of homozygosity analysis and haplotype mismatch rate:** A multisample VCF file for the Ingush and Chechen cohort was generated using GLnexus [27]. Genotype-level filtering was applied: genotypes with DP < 10 or GQ < 30 were set to missing (no-call). A subset of 328 unrelated individuals was selected based on plink2 (v. 2.00a5) KING [28,29] kinship coefficients < 0.0625. Runs of homozygosity (ROH) were identified using bcftools/ROH (v. 1.13) [30]. Only biallelic single-nucleotide variants with a call rate > 95% were included in the analysis. The *SLC19A2* c.1223+1G>A–associated haplotype was defined as the shared homozygous interval encompassing the variant and present in all homozygous patients (P3–P10). Haplotype concordance among patients was quantified using a custom Python3.11.9 code (https://github.com/SergeiFomenko/code-for-Role-of-SLC19A2-c.1223-1G-A-founder-effect...-article-, accessed on 10 May 2026). For heterozygous carriers and non-carriers, mismatch rates relative to the consensus patient haplotype were calculated across the defined interval as the proportion of discordant genotypes. A genotype was considered discordant if it differed from the homozygous reference state of the patient haplotype (i.e., 0/0 vs. 1/1 or 1/1 vs. 0/0).

**Principal component analysis:** Genotype data were subjected to linkage disequilibrium (LD) pruning prior to analysis to reduce marker redundancy. The resulting variant set was transformed using Patterson scaling. Principal component analysis (PCA) was then performed on the scaled genotype matrix using a singular value decomposition (SVD)-based approach as implemented in the scikit-learn Python library. PCA was computed on the transposed matrix (individuals × variants), the proportion of variance explained by each component was recorded.

**Interpretation of the pathogenicity of genetic variants:** Interpretation of the pathogenicity of genetic variants was performed according to the ACMG guidelines [31].

## 5. Conclusions

Mapping new endemic regions for hereditary conditions, such as the discovery of TRMA clustering in Ingushetia, offers opportunities for early intervention and personalized medicine, encourages community engagement in research, and can inform policy with respect to rare disease management. Furthermore, such studies facilitate collaboration between clinicians, geneticists, historians, and policymakers, fostering interdisciplinary efforts to address public health needs in underserved areas.

## Figures and Tables

**Table 1:** Clinical characteristics of patients.

	Patient 1	Patient 2	Patient 3	Patient 4	Patient 5	Patient 6	Patient 7	Patient 8	Patient 9	Patient 10
**Ethnic origin**	Altai	Central Asia	Ingushetia	Ingushetia	Ingushetia	Ingushetia	Ingushetia	Ingushetia	Ingushetia	Ingushetia
**Gender**	male	female	male	male	male	male	male	female	female	male
**Consanguineous marriage**	no	yes	no	no	no	no	no	no	no	no
**Familial history**	-	Early death of affected sibling	-	Affected sibling (P5)	Affected sibling (P4)	Early deaths of two siblings	Early deaths of two siblings	-	-	Early death of affected sibling
**Initial diagnosis**	Bone marrow failure	TRMA	MDS	TRMA	-	Aplastic anemia? MDS?	MDS?	MDS? DIDMOAD syndrome?	TRMA	TRMA
**Onset age of symptoms**										
**Diabetes**	7 years	1.5 years	1.5 years	1 year	11 months	9 months	3 years	3 years	3 years	1.8 years
**Deafness**	no information of age of manifestation	8 months	1.8 years	2 years	not manifested	2.5 years	1 year	1 year	1 year	no information of age of manifestation
**Anemia**	4 months	3 months	1 months	10 months	11 months	n/a	n/a	1.5 years	3 years	1.8 years
							5 years—first blood count			
**Ophthalmic features**	-	-	-	Optic atrophy	-	Vision acuity impairment	Optic atrophy. nystagmus	Vision acuity impairment		-
				1.2 years			3 years			
**Cardiac features**	Mitral valve insufficiency	Patent ductus arteriosus	Supraventricular tachycardia	Supraventricular tachycardia	-	-	-	-	n/a	-
**Diagnosis of TRMA**	11 years	1.5 years	2 years	3 years	11 months	16 years	15 years	5 years	3.5 years	2 years
***SLC19A2*** **variant**	c.120C>G, c.584T>C	c.196G>T	c.1223+1G>A	c.1223+1G>A	c.1223+1G>A	c.1223+1G>A	c.1223+1G>A	c.1223+1G>A	c.1223+1G>A	c.1223+1G>A

## Data Availability

The data presented in this study are available on request from the corresponding author. The data are not publicly available due to privacy restrictions, as they contain sensitive genetic and clinical information that could compromise patient confidentiality.

## Abbreviations

The following abbreviations are used in this manuscript:

TRMA	Thiamine-responsive megaloblastic anemia
WGS	Whole-genome sequencing
THTR-1	High-affinity thiamine transporter 1
ROH	Runs of homozygosity
PCA	Principal component analysis
MDS	Myelodysplastic neoplasms
DIDMOAD	Diabetes insipidus, diabetes mellitus, optic atrophy, and deafness (Wolfram syndrome)
NMD	Nonsense-mediated decay
